# Synergistic Effect of Plant Compounds in Combination with Conventional Antimicrobials against Biofilm of *Staphylococcus aureus, Pseudomonas aeruginosa*, and *Candida* spp.

**DOI:** 10.3390/ph16111531

**Published:** 2023-10-30

**Authors:** Graziana Bonincontro, Sarah Adriana Scuderi, Andreana Marino, Giovanna Simonetti

**Affiliations:** 1 Department of Environmental Biology, Sapienza University of Rome, P.le Aldo Moro, 5, 00185 Roma, Italy; graziana.bonincontro@gmail.com; 2 Department of Chemical, Biological, Pharmaceutical and Environmental Sciences, University of Messina, Viale Ferdinando Stagno D’Alcontres, 98100 Messina, Italy; sascuderi@unime.it

**Keywords:** antimicrobials, *Candida* spp., microbial biofilm, plant compounds, *Pseudomonas aeruginosa*, *Staphylococcus aureus*, synergism

## Abstract

Bacterial and fungal biofilm has increased antibiotic resistance and plays an essential role in many persistent diseases. Biofilm-associated chronic infections are difficult to treat and reduce the efficacy of medical devices. This global problem has prompted extensive research to find alternative strategies to fight microbial chronic infections. Plant bioactive metabolites with antibiofilm activity are known to be potential resources to alleviate this problem. The phytochemical screening of some medicinal plants showed different active groups, such as stilbenes, tannins, alkaloids, terpenes, polyphenolics, flavonoids, lignans, quinones, and coumarins. Synergistic effects can be observed in the interaction between plant compounds and conventional drugs. This review analyses and summarises the current knowledge on the synergistic effects of plant metabolites in combination with conventional antimicrobials against biofilms of *Staphylococcus aureus*, *Pseudomonas aeruginosa*, and *Candida albicans*. The synergism of conventional antimicrobials with plant compounds can modify and inhibit the mechanisms of acquired resistance, reduce undesirable effects, and obtain an appropriate therapeutic effect at lower doses. A deeper knowledge of these combinations and of their possible antibiofilm targets is needed to develop next-generation novel antimicrobials and/or improve current antimicrobials to fight drug-resistant infections attributed to biofilm.

## 1. Introduction

Bacteria and fungi exist predominantly within biofilms in natural and clinical settings. Bacterial and fungal biofilms are a structured community of microbial cells adherent to a surface and enclosed in a self-produced polymeric matrix that plays an essential role in many human diseases. These are sessile microbial communities constructed by surface attachment of planktonic bacteria, followed by cell–cell interactions with extracellular matrix that develop into growing colonies having a complex three-dimensional structure, thereby contributing to chronic infections [1]. 

According to the National Institute of Health (NIH, USA), about 80% of all microbial infections are caused by biofilms [2]. Infections and diseases associated with biofilms include skin infections, chronic wounds, otitis media, vaginal infections, keratitis, and respiratory and urinary tract infections. *Staphylococcus aureus*, *Pseudomonas aeruginosa*, and *Candida* spp. are responsible for many types of chronic biofilm-associated infections [3]. 

*S. aureus* is an opportunistic Gram-positive bacterium which causes several human infections, including tissue infection (skin, respiratory tract, bone articulations, endocarditis) and invasive infection, which can lead to sepsis and toxic shock syndrome; *S. aureus* biofilm-associated infections are also involved [4].

*P. aeruginosa* is a Gram-negative opportunistic pathogen commonly found in soil and water [5]. *P. aeruginosa* infections are mostly related to the presence of bacterial biofilm and include keratitis and respiratory mucosal and urinary tract infections [2]. 

*Candida* spp. colonise the oral cavity and the gastrointestinal and genitourinary tracts. *Candida* spp. are fungal pathogens responsible for superficial mucosal infections and for the majority of the reported global deaths by fungi in both immunocompromised and immunocompetent individuals [6]. *Candida* is implicated in biofilm-associated infections such as skin, oral, and vaginal infections and infections involving implanted medical devices. 

Nowadays, antibiotics and antiseptics appear to be less active against bacterial and fungal infections characterised by the presence of biofilm, for reasons mostly attributable to poor penetration of the extracellular matrix [7].

Therefore, the research of new compounds or alternative therapeutic approaches to combat biofilm represents a critical concern. Studies have demonstrated the antibiofilm effects of several natural compounds such as phenols, essential oils, terpenoids, lectins, and alkaloids, revealing their abilities to not only inhibit biofilm formation but destroy mature biofilm structures too [8,9].

The purpose of this article is to review the current knowledge concerning the antibiofilm property of phytochemicals with antimicrobial properties in combination with antibiotics and synthetic antimicrobials against *S. aureus*, *P. aeruginosa*, and *Candida* spp., with a view to their future application for the control of these microorganisms.

Synergism activity studies published between 2011 and 2023 were selected as a result of internet database searches, such as Google Scholar, PubMed, and Sciencedirect, using the following keywords: “*Staphylococcus aureus* biofilm”, “*Pseudomonas aeruginosa* biofilm”, “*Candida* biofilm”, “chronic infections”, “resistance”, “synergism”, “natural compounds”, “antibacterial drugs”, “antifungal drugs”, “antibiofilm”, and “polymicrobial biofilm”.

## 2. Biofilm of Bacteria and Yeasts

Biofilms are communities of microorganisms in nature that are attached to a biological or abiotic surface and are surrounded by a self-generated extracellular matrix mainly composed of polysaccharides, secreted proteins, and extracellular DNA [10,11]. A biofilm can consist of a single microbial species or a combination of different species in the microbial composition of their community and microenvironment. Biofilm growth allows bacteria and fungi a protected lifestyle that withstands hostile environmental conditions [12]. Furthermore, biofilms protect microorganisms from the host’s immune system and also increase their resistance to conventional antimicrobials by approximately 1000-fold [13,14,15].

Chronic *S. aureus*, *P. aeruginosa*, and *Candida* species infections associated with biofilms have become increasingly difficult to treat with current antimicrobials [16,17,18,19].

### 2.1. Steps of Biofilm Formation

The steps leading to microbial biofilm formation are dynamic and complex [12,20,21,22]. The biofilm development process includes the initial adhesion of microorganisms onto a surface, reversible attachment to the substratum, irreversible attachment and colonisation, aggregation and expansion, biofilm maturation, and finally biofilm dispersal and controlled detachment [15].

#### 2.1.1. Adhesion

The initial reversible adhesion constitutes the serendipitous meeting between a surface and a planktonic microorganism [23]. Microorganisms begin biofilm formation due to specific outer pressure, such as nutrition, extreme pH, extreme temperature, UV radiation, high salt concentrations, desiccation, high pressure, or antibiotic treatment. It initiates with the favourable interaction between planktonic cells transported to the surfaces by sedimentation, convection, Brownian motion, or hydrodynamic forces [11]. This primary adhesion is achieved through the effects of electrostatic forces, Lifshitz–van der Waals interactions, and hydrophobic interactions [23,24].

The irreversible attachment is attained with the involvement of bacterial and fungal structural adhesins [25]. Bacterial adherence structures, including pili/fimbriae, non-fimbrial adhesions, and flagella, were identified to be involved in the development of biofilms [26].

The biofilms of *S. aureus* can be classified into ica-independent and ica-dependent biofilms. The ica-independent biofilms are more frequently found in methicillin-resistant *S. aureus* (MRSA) isolates. These biofilms involve proteins belonging to the family of microbial surface component recognizing adhesive matrix molecules (MSCRAMMs), such as fibrinogen and fibronectin-binding proteins FnBPA and FnBPB, and *Staphylococcus* protein A (Spa), *S. aureus* surface proteins C and G (SasC and SasG), clumping factor B (ClfB), extracellular adherence protein (Eap), and biofilm-associated protein (Bap). The ica-dependent biofilms are associated with exopolysaccharide intercellular adhesin/poly-N-acetylglucosamine (PIA/PNAG), encoded by the icaADBC operon, involved in intercellular adhesion [27,28].

*P. aeruginosa* cells begin irreversible attachment by making strong adhesive forces and aligning their long axes parallel to the surface. During infection, the type IV pili, potentially, can initiate the adhesion between *P. aeruginosa* and the host surface [29]. The type IV pili bind the glycolipids asialo-GM1 and asialo-GM2 on epithelial cells of the surfaces [30]. Functional amyloid fibrils (Fap), which provide mechanical robustness to the biofilm, increase adhesion and cell aggregation [31].

*Candida* adhesins, which are cell wall proteins, play a pivotal role in this process by facilitating attachment to various cells, including epithelial cells and other microorganisms, as well as abiotic surfaces, through the binding to specific amino acids or sugar residues. In the case of *C. albicans,* numerous adhesins are part of the Als (agglutinin-like sequence) family. Similar ALS-like proteins have also been identified in *C. parapsilosis, C. tropicalis, C. dubliniensis, C. lusitaniae,* and *C. guilliermondii* [32]. *C. albicans* Eap1 participates in adhesion to polystyrene substrates; Hwp2, Pga1, and members of the Sap family play direct or indirect roles in adhesion to human cell lines or in cellular aggregation [33]. Recently, Thierry Mourer and colleagues have demonstrated that amyloid protein Pga59 is used by the fungal cell to mediate cell–substrate interactions and biofilm formation. The Pga59 cell wall protein is an amyloid-forming protein involved in adhesion and biofilm establishment in the pathogenic yeast *C. albicans* [34].

#### 2.1.2. Irreversible Adhesion and Aggregation

A cell-to-cell signalling mechanism referred to as quorum sensing (QS) also coordinates the initial formation of microbial biofilms [35].

Both Gram-negative and Gram-positive bacteria employ QS mechanisms to regulate biofilm formation by releasing signalling molecules (autoinducers, AIs) [36]. Gram-negative bacteria primarily produce acyl homoserine lactones (AHLs), whereas Gram-positive bacteria use oligopeptides [37,38,39]. In *C. albicans* QS, the best-known quorum-sensing molecules are farnesol, farnesoic acid, and tyrosol [40].

The irreversible adhesion of bacteria is progressed through the production of exopolysaccharides (EPSs) regulated by QS. The main constituents of EPSs, including polysaccharides, proteinaceous pili/fimbriae, other protein components, lipids, DNAs, and other compounds, depend on the species and the environmental factors [41,42,43,44,45]. Secondary messenger c-di-GMP is considered as one of the incentives for the transition from reversible to irreversible adhesion through EPS production and the structures of the cell’s surface [46]. EPS constituents are most often stabilised by intermolecular networks which involve specific binding proteins or networking between ECM constituents. Overall, EPS plays serious roles in adherence to surfaces, signalling, cell–cell recognition, biofilm formation, formed biofilm, protection of cells, retention of water, and genetic exchange [43,45,47,48]. EPS secretion continues till the third step of biofilm production to ensure the protected adhesion of bacteria and fungi to the surfaces.

The *S. aureus* auxiliary gene regulator (arg) QS system has a role in controlling the development of biofilms*. agrB*, a gene associated with the secretion of virulence factors, stimulates biofilm dispersion through increased production of the SplABCDEF serine proteases and the Aur metalloproteinase. PSMs have surfactant-like characteristics that are involved in the dispersion of biofilm. Other systems such as TCSs ArlRS, SaeRS, and SrrAB in *S. aureus* regulate virulence genes and biofilm formation. In *S. aureu*s, a subpopulation of cells undergoes lysis to release extracellular DNA that glues together the ECM. The holin-like CidABC and anti-holin-like LrgAB systems control this process [27]. CidA facilitates the release of genomic DNA and the production of biofilms via controlling the actions of murein hydrolases. The LrgAB, regulated from protein LytSR TCS, prevents CidA-mediated lysis [27].

*P. aeruginosa* cells produce EPS and form clusters following the early attachment steps. EPS is made up of lipids, proteins, nucleic acids, and polysaccharides [49]. The exopolysaccharides’ composition includes alginate, polysaccharide synthesis locus (Psl), and pellicle polysaccharide (Pel). Alginate is mainly produced by *P. aeruginosa* clinical isolates originating from the lungs of cystic fibrosis (CF) patients. It is a linear polymer consisting of β-D-mannuronic acid and α-L-guluronic acid, controlled by the algACD operon, and has an important role in the protection and structural stability of biofilm [19]. Psl is a neutral polysaccharide composed of a repeating pentasaccharide, consisting of D-mannose, D-glucose, and L-rhamnose, mainly involved in the initial attachment of cells and early biofilm formation, whereas Pel is a cellulose-sensitive exopolysaccharide composed of 1→4 linked partially acetylated galactosamine and glucosamine sugars, essential for late-stage biofilm formation and maturation [49,50,51,52,53]. Moreover, CdrA accessory proteins also exist in biofilm matrices [54]. *P. aeruginosa* also produces two small soluble lectins, LecA and LecB, which bind to the repeating sugar unit in the exopolysaccharide and help *P. aeruginosa* adhere to targets in the host organism [55,56,57]. Extracellular DNA (eDNA)-dependent matrix is a critical component for several functions that maintain the stability of biofilm and the survival of bacteria in this environment. eDNA is released by cell death and lysis, which mainly occur in the interior of biofilms (e.g., the stalk of the mushrooms). eDNA promotes cell–cell adhesion and biofilm self-organisation and enables the formation of a stable biofilm structure [58]. In *P. aeruginosa*, eDNA overcomes the electrostatic repulsion between negatively charged polysaccharide strands by binding to Ca^2+^ to produce “cationic bridging” [25,59,60,61]. The cationic bridge promotes cell aggregation, enhances *P. aeruginosa* biofilms, and aids in initial attachment to foreign surfaces. Additionally, eDNA can interact with Pel and Psl to create fibre-like networks, which is likely to increase the biofilm’s resilience [62,63,64].

Once yeast cells have started to adhere to each other and to a surface, the transitional stage or morphological modification, where yeast cells change into hyphae via the transcriptional regulators Bcr1, Brg1, Efg1, Tec1, Ndt80, and Rob1, occurs. Hwp1 is required for hyphal development, mating, and the maintenance of biofilm integrity.

In the context of polymicrobial biofilms, the hyphae contribute to the architectural stability of the biofilm and act as a support for yeast cells, pseudohyphae, and other microorganisms [65]. During cell proliferation, yeasts accumulate an extracellular matrix (ECM) [66]. There are four main macromolecular ECM constituents: proteins (55%), carbohydrates (25%), lipids (15%), and nucleic acid. There are more proteins identified in the biofilm matrix, including glycolysis enzymes that may degrade extracellular biopolymers acting as a source of energy. In *C. albicans* biofilm, the dominant polysaccharide in the ECM is the mannans glucan complex, namely, α-1,2 branched α-1,6 mannan, which is associated with linear β-1,6 glucan and a small amount of β-1,3 glucan. The profile lipid is composed of glycerolipids (99.5%), with fewer sphingolipids (0.5%). In addition, the only sterol detected was ergosterol, though at low concentrations. EPS gives a three-dimensional structure to the formed biofilm in which the cells may stay immobilised. In *C. tropicalis*, glucose and hexosamine are the major carbohydrates present. Small amounts of proteins, uronic acid, and phosphorus may also be present [32]. Another key contributor to the stability and growth of *C. albicans* biofilms in the ECM is eDNA with predominantly random non-coding sequences. eDNA is likely be released via autolysis [55]. The ECM is partially self-produced and secreted by *C. albicans* cells within the biofilm but may also contain aggregates of *C. albicans* cells and lysed host cells, as well as epithelial cells and neutrophils. Two transcription factors involved in regulating ECM production for *C. albicans* biofilms are Zap1 and Rlm1 [67,68,69].

#### 2.1.3. Colonisation and Maturation

Bacteria have minimal metabolic activity at deeper levels of mature biofilms. However, specific channels permit the exchange of nutrients and products between microorganisms enclosed within the biofilm and the outer environment, which favours the colonisation and maturation of the microorganisms [48]. Once microorganisms have irreversibly attached to a surface, the process of biofilm maturation begins. Bacteria continue to reproduce using the AIs signals within the embedded EPS matrix, and this leads to the formation of microcolonies and the maturation of biofilms [22,46]. During the process of maturation, the gene expression pattern of the sessile cells differs significantly from that of the planktonic cells; motility within the microcolonies, such as the production of bacterial surface structures, is restricted. Bacteria within deeper areas of mature biofilms possess lower metabolic activity. The minimal availability of nutrients and hypoxic conditions of the environment have been recognised as factors that stimulate the transition of bacteria in persister cells, a subpopulation of dormant phenotypic variants, detected in biofilm chronic infections. In staphylococcal biofilms, persister cells comprise a subpopulation of cells highly tolerant to antibiotics without being genetically resistant [70].

Yeasts produce microcolonies, and the coalescence of microcolonies yields the basal level of the biofilm. In the final maturation step, there is a wide accumulation of extracellular matrix material [71]. Persister cells also form, and although they make up only a small part of the biofilm population, these cells are associated with cases of tolerance to antimicrobial treatments [65]. *C. albicans* mature biofilms exhibit a dense network composed of blastophores and hyphae surrounded by ECM [71].

#### 2.1.4. Dispersion

After the maturation of biofilm, the microorganisms detach and disperse to start a new biofilm community [12]. The biofilm detachment process, also known as the dispersion stage, represents the final process of biofilm development.

It is regarded as a strategy of bacterial cells to detach from the biofilms and begin another biofilm life cycle [72]. The biofilm dispersal process is regulated by environmental signals (oxygen, nutrients, temperature, and signalling molecules), the intracellular reduction in the concentration of c-di-GMP, and the upregulation of motility or QS genes, though many bacterial dispersal signals remain cryptic. The dispersion mechanisms differ among bacteria; however, the process can be divided into three common steps: escape of cells from the microcolonies, movement of freed cells, and attachment of the cells to another substrate [73,74].

During the biofilm dispersal phase, yeast cells disperse non-adherent budding daughter cells to make new communities [32,75]. The detachment of cells from biofilms involves mainly yeast cells, not filaments. The released cells display higher levels of adhesion to plastic or endothelial cells, probably due to their increased propensity to produce hyphae. In addition, the released cells were phenotypically distinct and more virulent than planktonic cells in a disseminated infection model. Thus, biofilm dispersion yields a unique class of yeast cell with increased ability to create new biofilms and cause infection [71,75].

The dissemination and colonisation of new target substrates explain recalcitrant microbial chronic infections. Moreover, biofilms protect the invading microorganisms against the immune system of the host via the impaired activation of phagocytes and the complement system [76].

Consequently, biofilm-associated diseases are persistent infections that are slowly developing, rarely resolved by the immune system, and refractory to antimicrobial treatments.

## 3. Bacterial and Fungal Resistance

### 3.1. Bacterial Biofilm Resistance

It is believed that biofilm-related organisms account for more than 65% of all microbial infections and exhibit high resistance to antimicrobial agents and components of the host defence system (both innate and adaptive) [77]. Within the biofilm, the bacteria adapt to environmental anoxia and nutrient limitations, showing an altered metabolism and altered gene expression profile, concomitant with lower metabolic activity, reduced cell proliferation, and increased nutrient sequestration. In addition, biofilm growth is associated with an increased level of mutations, and the proximity of the biofilm-embedded cells facilitates the horizontal transfer of resistance genes between the bacteria [27]. The principal concern with biofilm-related infections involves the difficulty in fully eradicating the infection, despite aggressive antimicrobial therapy. There are two primary mechanisms that explain how bacterial pathogens can evade antimicrobial therapy: resistance and tolerance. Resistance, which is measured by the minimum inhibitory concentration (MIC), refers to the ability of bacteria to multiply despite treatment with an antimicrobial compound. Resistance is typically inherited from mother bacteria or acquired from horizontal gene transfer, which is facilitated by the close proximity of bacterial cells within a biofilm. Resistance can be mediated by several processes, including drug inactivation or modifications, alteration of the binding site, reduced drug accumulation through increased efflux or decreased entry, and use of alternative metabolic pathways. Tolerance, by comparison, is defined as the capability of a microbe to survive despite treatment with a bactericidal antibiotic to which it is susceptible based on the minimum inhibitory concentration value [7].

In *S. aureus* infections, a significant problem is the rapid development of antibiotic resistance associated with biofilms [18]. Cell heterogeneity can exist in staphylococcal biofilms due to the development of persister cells through the acquisition of antibiotic resistance genes through horizontal gene transfer [78,79]. Among the various mechanisms by which this complex phenomenon may occur, those involving antibiotic efflux, enzyme activity, and reduced permeability are noteworthy [18]. The treatment of biofilm infection requires sensitive and well-penetrating antibiotics to ensure a sufficient concentration of effective antibiotics at the site of the biofilm infection. Bacterial cells within a biofilm are likely to encounter subinhibitory concentrations of antibiotics, which were shown to potentially stimulate biofilm production and alter the composition of the biofilm matrix [70]. Hence, tetracyclines, macrolides, rifamycins, lincosamides, quinolones, fusidic acid, oxazolidinones, sulfonamides, and nitroimidazole are preferred to glycopeptides, aminoglycosides, polymyxins, and β-lactamases because they can penetrate deeper [18]. The secretion of β-lactamase from a biofilm-embedded bacteria into the biofilm matrix can prevent the β-lactam antibiotics from acting on a neighbouring cell even if the latter does not produce the enzyme [27].

In chronic *P. aeruginosa* infections, the typical biofilm lifestyle significantly diminishes the effectiveness of antimicrobial treatments, as it results in inherent tolerance. This tolerance encompasses physical and physiological factors, along with biofilm-specific genes that can temporarily shield against antibiotics, thereby fostering the emergence of resistance [80]. The negatively charged polysaccharides (especially the Pel polysaccharide and alginate of *P. aeruginosa*) can effectively sequestrate the positively charged aminoglycoside class of antibiotics, such as tobramycin, thus preventing them from penetrating the deeper layers of the biofilm [81]. The eDNA through chelating cations such as magnesium ions forms a cation-limited environment that results in the induction of the PhoPQ and PmrAB TCSs in *P. aeruginosa*. These TCSs regulate cationic antimicrobial peptide resistance by upregulating the PA3552-PA3559 operon. The DNA-induced expression of PA3552-PA3559 results in up to a 2560-fold increase in the resistance to cationic antimicrobial peptides and a 640-fold increase in the resistance to aminoglycosides [82]. Biofilm-associated *P. aeruginosa* expresses the gene ndvB, which encodes a glycosyltransferase that catalyses the synthesis of periplasmic β-(1→3)-cyclic glucans. The glucans are thought to promote aminoglycoside resistance by sequestering the antibiotics (e.g., tobramycin) in the periplasm away from their cellular target. When *P. aeruginosa* biofilms are exposed to β-lactam antibiotics or colistin, various resistance mechanisms are induced, such as the increased expression of β-lactamase and the production of modified lipopolysaccharides, which make the bacteria resistant to colistin and other polymyxin antibiotics. tssC1, which is implicated in type VI secretion (T6S), is upregulated in *P. aeruginosa* biofilms. The upregulation of tssC1 is important for the induction of biofilm-associated antibiotic resistance to tobramycin, gentamicin, and ciprofloxacin. The expression of RND efflux pumps and genes involved in type III secretion were upregulated in antibiotic-resistant biofilms of *P. aeruginosa* that have developed in the presence of azithromycin [27].

### 3.2. Candida Biofilm Resistance

Biofilm, a self-protected life mode of the *Candida* species [16,17], is the main cause of antibiotic-resistant *Candida* infections. Biofilm-associated infections are hard to treat and constitute a challenge for clinicians because of their persistence and resistance to the majority of known antifungal drugs. *Candida* is able to form biofilms both on biotic and abiotic surfaces, for example on mucosae and medical implants.

Biofilm resistance is multifactorial and mechanistically complex and uses both methods akin to traditional, planktonic antifungal resistance as well as mechanisms specific to the biofilm lifestyle [17]. However, it can be mainly attributed to three features: production of an extracellular matrix; drug efflux pump overexpression, and persister cell presence [83].

In total, 55% of the extracellular matrix is made up of proteins, followed by 25% of carbohydrates, 15% of lipids, and 5% of extracellular DNA, and it plays a major role in *Candida* biofilm resistance [83]. Its two components, extracellular DNA and β-glucan, increase biofilm resistance to different antifungals in an unclear way; this may also entail reduced drug penetration and a matrix–antifungal sequestration mechanism [84]. It is also believed that the extracellular matrix plays a role in the preservation of nutrients, water, and enzymes [85,86]. The formation of biofilm matrix β-1,3 glucan, biofilm adhesion, and resistance to azole treatments are all hampered when this matrix delivery pathway is disrupted.

The efflux pumps are mainly involved in resistance mechanisms during the early biofilm developmental phase [87]. With transcriptional analysis, it has been possible to show the greater expression of efflux pump genes following 12 h of biofilm formation in contrast to 48 h of full biofilm formation [88]. These findings further support the theory of time-specific efflux pump functionality [88]. *C. glabrata* and *C. tropicalis* biofilms are also characterised by the upregulation of efflux pumps [89,90].

Furthermore, at the later stages of biofilm growth, the content of ergosterol in the cell membranes of biofilm cells is much lower than that of planktonic cells [17]. This reduced presence of ergosterol may impair the effectiveness of medications that target ergosterol, such amphotericin B and azoles.

Namely, alterations in the transcriptional profile of sterol pathway genes have been shown, examining the various phases of biofilm formation [91,92]. Through microarray analysis, Nett and colleagues have demonstrated that an increased transcription of *ERG11*, which codes for the azole drug target, and ERG25, which codes for a putative C4 methyl sterol oxidase, may be involved in the C4-demethylation of intermediates in ergosterol biosynthesis [92].

*Candida* biofilm is also characterised by the presence of a subpopulation of cells, called persister cells, metabolically dormant variants of microbial deep-seated biofilm-forming microbes that are resistant to a variety of medications, such as amphotericin B, azoles, and chlorhexidine [17]. They form only upon adherence to a surface [83].

Persister cells are present within both *Candida* and bacterial biofilms [93]. *C. albicans* and *C. krusei* biofilms frequently possess persister cells, whereas *C. glabrata* spp. do not [93,94].

General stress response upregulation, cell density, and quorum sensing are the main features which allow biofilm-specific resistance mechanisms [83].

The greater number of fungal cells in a biofilm as opposed to planktonic development seems to be responsible for drug resistance [95,96]. Perumal and Chaffin [95] demonstrated that both planktonic and biofilm cells revealed higher azole drug resistance at higher than at lower cell densities, indicating that antifungal resistance is influenced by high biofilm cell density.

*Candida* biofilm cell density is strictly linked with quorum sensing. *C. albicans* produces two main quorum-sensing molecules, tyrosol and farnesol, which have conflicting effects [97,98]. It has been demonstrated that farnesol prevents the development of biofilms and the yeast-to-hyphae transition [85,98], and it seems to negatively regulate drug resistance. This effect has been observed for *C. albicans* and *C. dubliniensis* biofilms [99,100].

In addition, greater stress responses to biofilm presence can influence drug susceptibility and promote drug resistance [83]. Uppuluri and colleagues identified calcineurin, a Ca^2+^ calmodulin-activated serine/threonine-specific protein phosphatase, crucial for morphogenesis, virulence, and homeostasis, as being involved in biofilm resistance [101]. Hsp90, one of the heat shock proteins that stabilises several host proteins, constitutes another stress response pathway contributing to *Candida* biofilm resistance [102].

## 4. Antimicrobial Activity of Plant Compounds

For 350 million years, plants have survived various environmental stresses, both of abiotic and biotic origin [103]. Environmental (abiotic) factors can be considered as the lack of nutrients and oxygen, drought, salt, unfavourable temperature swings, intense sunshine, and conditions brought on by human activity, including pollution, pesticide use, and elevated UV radiation [104,105]. Herbivore pests, nematodes, bacteria, fungus, viruses, and insects are examples of biotic influences. [106]. To withstand such assaults, plants detect pathogens and initiate immune responses, such as fortifying the cell wall, synthesising lytic enzymes, and producing pathogenesis-related proteins [107].

Moreover, plants produce a broad range of compounds that constitute a defence mechanism against antagonistic biotic and abiotic stresses [103]. They are called ‘secondary metabolites’ because they are products of secondary plant metabolism and are biologically dynamic.

These compounds may exist in a constitutively stored, inactive state within plant cells or on the plant’s surface before encountering microorganisms, or they can be synthesised after infection from existent forerunners. Alternatively, their biological synthesis can be induced in reaction to an infection [108].

Phytoalexins are the inducible compounds and include terpenoids, glycosteroids, flavonoids, and polyphenols [103].

Secondary metabolites not only improve the defence against biotic and abiotic stressors but also provide a substantial supply of antiviral, antifungal, antimalarial, antibacterial, antioxidant, anti-inflammatory, anticancer, antidiabetic, and immunosuppressive compounds [109,110]. Secondary metabolites are also nutritional constituents that can improve the physiological and cellular activities in animals and humans that consume them [111].

The use of plants as therapies in traditional medicine is nowadays well established in several diseases. The most important advantages linked with the use of herbal plants are their safety, including reduced cost-effectiveness in comparison to better patient tolerance and fewer unwanted side effects, sustainability, improved biodegradability, and easy availability [112].

Plants enriched in natural secondary metabolites may be a promising approach to the discovery of potential alternative therapeutics to combat antimicrobial resistance. Specifically, plants have been widely documented as capable of producing drug resistance inhibitors that facilitate the delivery of antimicrobial compounds [112].

Different antimicrobial mechanisms have been very well recognised: the degradation of the proton driving force with ion leakage, natural action of the cell components, disruption of the outer membrane of Gram-negative microorganisms through the release of lipopolysaccharides, interaction with membrane proteins, such as ATPases and other enzymes, and inhibition of enzyme synthesis [113].

Regarding antibiofilm activity, the possibility of using natural compounds that can inhibit quorum sensing has been proposed to treat biofilm-associated infections [27].

Numerous secondary plant metabolites have been demonstrated to possess anti-quorum and antibiofilm properties, despite typically lacking direct antibacterial activity [103,114]. Examples are halogenated thiophenones, which can manage the growth of biofilms [115]; baicalin hydrate and cinnamaldehyde target *P. aeruginosa*’s acyl-homoserine lactone-based quorum sensing system [116]; hamamelitannin targets *S. aureus*’s peptide-based system; and baicalin improves *P. aeruginosa*’s susceptibility to tobramycin [116].

Sesamin and Sesamolin, two lignans separated from the plant *Sesamum indicum* (L.), have been shown to stop previously infected worms from becoming infected in a *P. aeruginosa* infection model in *Caenorhabditis elegans*. The mechanism seems to be their capacity to attenuate the quorum sensing-regulated virulence factors of the bacteria [117].

However, there are no plant-derived antibiotics, although plants fight infections successfully, probably thanks to a successful defence mechanism they developed, based on “synergy” between the several secondary metabolites they synthesise [103].

Combination therapy is one method that can be used to increase the efficacy of antimicrobial therapy for difficult-to-treat infections, such as biofilm-related infections, given the dearth of new antimicrobial drugs with novel mechanisms of action and the rise in infections brought on by resistant microorganisms [118].

This strategy focuses on using natural enhancers to boost the antimicrobial medicine’s antibiofilm activity at concentrations where the antibiotic previously exhibited no antibiofilm activity. Thus, researchers have also been interested in how phytocompounds interact with antibiotics or chemotherapeutic antimicrobial agents. Numerous studies have shown that these phytocompounds exhibit promising synergistic effects when combined with these medications, offering benefits for treatment such as a wider range of efficacy, enhanced safety and tolerability, decreased toxicity, and decreased resistance to antimicrobial agents [119].

Therapeutic interactions are described as synergistic, indifferent, and antagonistic. This interaction is defined by the fractional inhibitory concentration index (FICI) [120]. The FICI is a non-parametric model defined by the following equation:

Σ FIC = FIC A + FIC B

where FIC A = MIC of drug A in combination/MIC of drug A alone; FICB = MIC of drug B in combination/MIC of drug B alone [121].

The following values obtained from the FICI equation establish the interaction relations: values ≤0.5 indicate synergistic interactions, values of 4.0 indicate antagonistic effects, and values between these two indicate no interaction [120].

## 5. Synergy between Antibacterial Chemotherapeutics and Natural Compounds against *S. aureus*

Biofilm formation adds to the resistance phenotype of the microbial pathogens towards host defence and conventional drug therapy, often resulting in serious and persistent infections [122]. Thus, a multifaceted approach is needed to combat this problem by discovering novel antimicrobial agents and/or new methodological strategies [123,124]. In the last decade, the interest in medicinal and aromatic plants and their components has remarkably increased.

Accordingly, Shinde and colleagues [125] focused on the effect of epigallocatechin gallate (EGCG), a polyphenol/catechin of green tea. The chemical structure features of EGCG include multiple hydroxyl groups and a gallate moiety. EGCG is prominently found in green tea (*Camellia sinensis*) leaves, accounting for a significant portion of their polyphenol content. EGCG exerts antioxidant, anti-inflammatory, anti-tumour, and antimicrobial effects on various Gram-positive and Gram-negative bacteria [125]. The antimicrobial effects of EGCG are exerted through several mechanisms, including cell membrane injury, enzyme inhibition, and the impairment of fatty acid biosynthesis. EGCG compromises cell wall integrity by interfering with the polysaccharides that make up the glycocalyx and bind peptidoglycan, affecting the biofilm attachment to the surface. Recently, reports hypothesised that the synergism between EGCG and several antibacterial substances can counteract bacterial biofilm by deteriorating the extracellular matrix elements and favouring the antibiotics’ action [126]. In this context, Shinde and colleagues demonstrated that a modified lipid-soluble EGCG, epigallocatechin-3-gallate-stearate (EGCG-S), in combination with tetracycline, is able to inhibit 94% of *S. aureus* ATCC 14990 biofilm formation [125]. Several studies demonstrated that EGCG impairs the assembly of phenol-soluble modulins (PSMs), Bap formation, and the integrity of the cell wall, hypothesising that this polyphenol might influence the cell adhesion to the surface. The potential of EGCG as a synergistic agent with antibiotics could be due to breaks in the extracellular matrix components, which allow antibiotic permeation, favouring their effect against biofilm [125,126].

Another natural compound drawing great interest is baicalein, which is an extract of *Scutellaria baicalensis* with antibacterial activity. A study by Chen and colleagues revealed baicalein’s antibiofilm properties using clinically isolated strains of *S. aureus* 17546 (t037). The molecular mechanism of its antibiofilm activity was studied also in combination with antibiotics such as vancomycin (VCM), showing its synergic effect [127]. It has been demonstrated that baicalein was able to prevent *S. aureus* biofilm development also at subinhibitory concentrations. Although baicalein (32 µg/mL) and VCM (4 µg/mL) alone did not exert any effect on bacterial counts in established biofilms, their combination was able to trigger a dramatic effect on the dispersal step. The authors demonstrated that baicalein could inhibit *S. aureus* biofilm formation by downregulating the quorum-sensing system regulators, like *agrA*, RNAIII, and *sarA*. The synergism between baicalein and VCM caused biofilm formation inhibition, the destruction of formed biofilm, and increased permeability to VCM [127].

**Figure 1:**
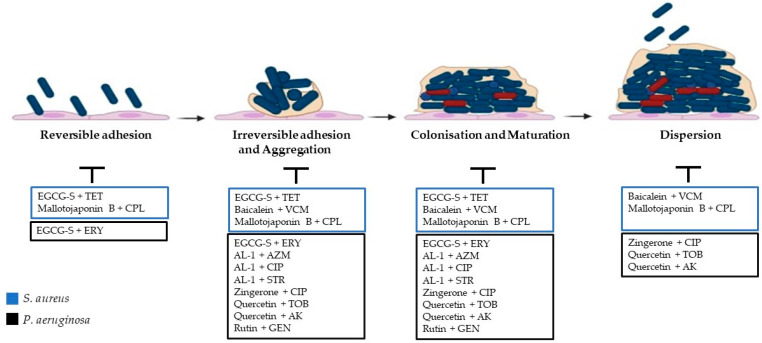
Activity of plant compounds in combination with antimicrobial drugs on different steps of bacterial biofilm formation. Regular cells are shown in blue; persister cells are shown in red. Epigallocatechin-3-gallate-stearate (EGCG-S), 14-alpha-lipoyl andrographolide (AL-1), erythromycin (ERY), tetracycline (TET), vancomycin (VCM), chloramphenicol (CPL), azithromycin (AZM), ciprofloxacin (CIP), streptomycin (STR), tobramycin (TOB), amikacin (AK), gentamicin (GEN).

Mallotojaponin B, a phenolic compound obtained from *Mallotus oppositifolius*, possesses several antibacterial effects. Nguena-Dongue and colleagues investigated the combinatory effect of mallotojaponin B with chloramphenicol against methicillin-resistant *S. aureus* ATCC 33591. Mallotojaponin B, belonging to the phenol family, exerts its effects through the alteration of membrane structural components or the inactivation of cell essential functions. Moreover, mallotojaponin B is able to alter membrane permeability and integrity and modify several intracellular functions induced by the hydrogen bonding of phenolic compounds to enzymes. MRSA ATCC 33591, which is chloramphenicol-resistant, was susceptible to mallotojaponin B’s activity. Nguena-Dongue and colleagues investigated the effects of their combinations against MRSA. Interestingly, a synergistic effect (FICI 0.393) with MIC reductions in a range from 12.5 μg/mL (MIC) to 0.781 μg/mL (1/16 MIC) for mallotojaponin B and from 250 μg/mL (MIC) to 1.95 μg/mL (1/128 MIC) for chloramphenicol was demonstrated [128]. These results demonstrated that the combination of mallotojaponin B and chloramphenicol exerts bactericidal synergistic effects on MRSA through the alteration and destruction of the cell membrane and biofilms formed by MRSA, suggesting that this combination could be a potential strategy against methicillin-resistant *S. aureus* (Table 1; Figure 1).

## 6. Synergy between Antibacterial Chemotherapeutics and Natural Compounds against *P. aeruginosa*

In *P. aeruginosa*-associated infections, antibiotic resistance is mediated in part by surface-attached biofilms [133]. Their framework becomes a physical barrier to antibiotic penetration and provides an altered microenvironment for pathogen survival inside the host [133]. Thus, there is an urgent need for revamping the treatment measures to counter bacterial biofilm infections like *P. aeruginosa* infection as well as to control the emergence of highly virulent strains. Previous studies have focused on the antibiofilm potential of various plant natural compounds including phenols, essential oils, terpenoids, lectins, alkaloids, polypeptides, and polyacetylenes, demonstrating that they not only inhibit biofilm formation but also eliminate mature biofilm structures [129,133]. The combination of plant extracts and antibiotics represents a template for developing antibiofilm drugs against *P. aeruginosa* biofilm-associated infections [134]. Several active substances present in ginger (*Z. officinale*) such as gingerols, shogaols, paradols, gingerdiols, and zingerone have been widely used in traditional herbal medicine [129]. Some of these chemical compounds are found to be clinically effective for the treatment of various diseases, including *P. aeruginosa* infections [129]. According to this, it has been demonstrated that zingerone could be a potential antibiofilm agent by making the biofilm prone to antibiotic treatment and by altering its formation process, demonstrating a synergistic effect with antibiotics. When the biofilm was grown in the presence of zingerone (10,000 µg/mL), ciprofloxacin (0.06 µg/mL) showed a significantly high eradication efficiency because zingerone modulated the biofilm formation, making it thin [129]. Altered biofilms were more susceptible to ciprofloxacin; so, biofilm grown in the presence of zingerone was more easily eradicated by ciprofloxacin as compared to the normally grown biofilms [129]. Therefore, ciprofloxacin can better penetrate thinner biofilms in combination with zingerone, becoming more efficient at eradicating *P. aeruginosa* biofilms. The results of the study suggested that zingerone has potent antibiofilm inhibition and eradication activities which increase the antibiotic effect [129].

Another report demonstrated the synergistic effect of 14-alpha-lipoyl andrographolide (AL-1), obtained from *Andrographis paniculate*, on *P. aeruginosa*-associated biofilm when combined with azithromycin, ciprofloxacin, and gentamicin [130]. The report revealed the antibiofilm properties of AL-1 and its ability to sensitise the bacterium to several antibiotics through synergistic effects. The combinatory treatment between AL-1 and antibiotics was demonstrated to decrease the production of EPS and pyocyanin compared to antibiotics alone, suggesting that the synergism effect is due to the inhibition of biofilm synthesis. The results have proven that AL-1 could be an effective compound to counteract *P. aeruginosa* infection when used in association with the most common antibiotics [130].

Rutin, a flavonoid isolated from the peels of *Citrus sinensis* with antimicrobial properties, has been studied for its antibiofilm potential against *P. aeruginosa*, in combination with the conventional antibiotic gentamicin [131]. The results showed that rutin exhibited an MIC at 800 μg/mL against *P. aeruginosa*. When the bacterium was treated with rutin (200 μg/mL) and gentamicin (2.5 μg/mL), the prevention of biofilm formation was improved in a synergistic manner [131]. Adherence and biofilm formation were attributed to EPS generation, which was similarly markedly inhibited in the tested strain, indicating a potential synergistic action in combination with gentamicin. The findings of the study confirm that rutin could be potentially used as an adhesion inhibitor to treat *P. aeruginosa* infections, displaying an enhanced antibiofilm property when combined with gentamicin. Even though the mechanism of inhibition of biofilm formation by rutin is unclear, the study indicates the potential use of rutin as a promising antibiofilm agent against *P. aeruginosa* as it induces ROS generation in bacteria, leading to oxidative stress and the death of bacteria [131]. Moreover, quercetin demonstrated interesting antibacterial effects too. The development of drug resistance in opportunistic pathogens represents one of the major healthcare challenges correlated with infection management [132]. Combination therapy has numerous advantages due to the simultaneous effect of two drugs on two separate cellular targets [132]. Nevertheless, the selection of the drugs should offer safety and a synergistic interaction against most of the strains. The efficacy of antibiotics in combination with quercetin, a natural flavonoid, against biofilm-forming *P. aeruginosa* strains previously isolated from catheter-associated urinary tract infections has been studied. Based on the antibiotic susceptibility pattern, the synergistic effect of quercetin (10 mg/mL) with selected antibiotics amikacin and tobramycin was tested by evaluating the MIC and FICI values. Quercetin was demonstrated to inhibit biofilm formation with an MIC value of 500 μg/mL; however, the combinations with the antibiotics amikacin and tobramycin were demonstrated to further inhibit biofilm formation, showing FICI values of 0.25 and 0.5, respectively. The effects of the synergistic combinations were further assessed with time–kill and biofilm cell viability assays. Quercetin and selected antibiotics affected biofilm formation and biofilm cell viability, showing 80% inhibition. These results demonstrated that synergistic combinations penetrate the matrix of the biofilm and cause the death of cells. In vitro infection studies showed that the drug combinations decreased the infection rate significantly by reducing the HEK-293T cell killing effect caused by *P. aeruginosa* [132]. A modified lipid-soluble EGCG, epigallocatechin-3-gallate-stearate (EGCG-S) (100 μg/mL), in combination with erytromycin (15 μg/mL), is able to inhibit 95% of *P. aeruginosa* ATCC-CRM-9027 biofilm formation, suggesting the potential of EGCG-S as a synergistic agent with antibiotics and as an antibiofilm agent [125]. It was demonstrated that EGCG influences the ability of Fap to form fibrils. Combinations with antibiotics favour the two agents’ penetration and their action on bacterial cells [135,136] (Table 1; Figure 1).

## 7. Synergy between Antifungal Chemotherapeutics and Natural Compounds against *Candida* spp.

Plants, in their ongoing battle for survival, have developed a wide range of defensive molecules to protect themselves from attacks by plant pathogenic fungi. These molecules, often present in plants as natural compounds, have demonstrated remarkable antifungal properties. In this context, numerous studies have explored the potential applications of these molecules in the fight against fungal infections, particularly those caused by *Candida* species. These investigations have focused on their synergistic interactions with synthetic antifungals, particularly in the context of biofilm-associated infections, offering an intriguing perspective for the development of effective antifungal therapies.

*Acorus calamus* Linn. is widely spread across Central Asia, North America, and Eastern Europe. Indian medicine uses this plant’s rhizome to treat several diseases like epilepsy, mental ailments, chronic diarrhoea, and dysentery and for the following symptoms and conditions: helminthiasis, amenorrhea, dysmenorrhea, nephropathy, calculi, strangury, hoarseness, flatulence, and dyspepsia [137]. α- and β-asarones, the primary bioactive substances, showed several bioactive features like antibacterial and antifungal properties [118,138,139,140,141]. The in vitro synergistic activity of α-asarone and β-asarone purified from *Acorus calamus* rhizomes and amphotericin B, fluconazole, and clotrimazole against *Candida* species has been reported by Kumar and colleagues.

α-asarone (8 μg/mL) combined with 0.03 μg/mL of amphotericin B reduces *C. albicans* biofilm by 36%. *C. tropicalis* biofilm is reduced by 39% using a combination of 0.25 μg/mL of amphotericin B and 4 μg/mL of α-asarone [118]. β-asarone showed a lower antibiofilm activity against both *C. albicans* and *C. tropicalis* when combined with amphotericin B. β-asarone (1 μg/mL) and amphotericin B (0.03 μg/mL) combined inhibited *C. tropicalis* biofilm by 21%. *C. albicans* biofilm is inhibited at the same rate by the combination of 8 μg/mL β-asarone and 0.06 μg/mL amphotericin B. It is evident from the results obtained by Kumar and colleagues that α-asarone and β-asarone had synergistic interactions also with fluconazole and clotrimazole. The MIC values decreased by eight times, and the inhibition of biofilms was significantly higher than with the individual compounds. α-asarone (8 μg/mL) and fluconazole (0.06 μg/mL) resulted in the most active combination against *C. albicans* with an inhibition rate of 40% of the biofilm formation [118]. β-asarone has been observed to suppress the morphogenesis and biofilm growth of *C. albicans* at subinhibitory doses [142].

The antifungal properties of plant-derived polyphenols are nowadays well known.

EGCG has garnered attention for its multifaceted biological activities, including its antifungal properties. In scientific research, EGCG has demonstrated inhibitory effects against various fungal species. It has been shown to inhibit the growth and yeast-to-hypha transition of *C*. *albicans* [143,144,145,146,147]. Several studies demonstrated that EGCG may synergise with fluconazole, amphotericin B, and ketoconazole against *C. albicans* [144,148]. Synergistic effects have been observed against all *Candida* spp. biofilm when EGCG was combined with amphotericin B by Ning and colleagues. *C. albicans* ATCC 10231 biofilm was reduced by 90% with 750 μg/mL of EGCG and 0.13 μg/mL of amphotericin B with a FICI of 0.19 [149]. The combination of 375 μg/mL of EGCG with 1.56 μg/mL of amphotericin B showed an inhibition of *C. parapsilosi*s biofilm of 90% with a FICI of 0.27. In addition, 3000 μg/mL of EGCG with 0.19 μg/mL of amphotericin B showed a 90% inhibition of *C. tropicalis* biofilm with a FICI of 0.19. The EGCG–amphotericin B combination resulted in a synergistic effect also against *C. krusei* (FICI = 0.31), *C. glabrata* (FICI = 0.31), and *C. kefyr* (FICI = 0.5). *C. krusei* is the most sensitive to this combination, with a sessile minimum inhibitory concentration 90 (SMIC90) of 187.5 μg/mL for EGCG and 0.16 μg/mL for amphotericin B [149]. In a study conducted by Behbehani and colleagues, the synergistic effects of EGCG in combination with fluconazole or ketoconazole against mature biofilms of *Candida* species strains were investigated [150]. Notably, no interaction was observed when EGCG was combined with fluconazole against two *C. albicans* strains (ATCC 24433 and a clinical isolate), as indicated by FICI values exceeding 0.5. However, in all other cases, the FICI values were below 0.5, signifying a synergistic interaction between EGCG and fluconazole or ketoconazole [150]. Similarly, Ning and colleagues investigated synergism between EGCG and fluconazole or miconazole. They performed an antibiofilm assay using *C. albicans* SC 5314, *C. albican*s ATCC 10231, *C. parapsilosis* ATCC 22019, *C. tropicalis* ATCC 13803, *C. glabrata* ATCC 66032, *C. kefyr* ATCC 46764, and *C. krusei* ATCC 14243. All the strains were found to be more sensitive to the EGCG in combination with fluconazole or miconazole. The concentrations needed to obtain biofilm inhibition were lower when used together. The combination of EGCG with fluconazole appeared to be additive rather than synergistic against *C. parapsilosis, C. krusei*, and *C. kefyr*, as indicated by the FICI value (FICI values of 0.56, 0.75, and 0.63, respectively), whereas the combination with miconazole had no synergistic effect only against *C. kefyr* (FICI = 0.63) [149]. This polyphenolic compound’s potential as an antifungal agent is attributed to its ability to target specific fungal components and pathways. Some authors have reported that the mechanism of action of EGCG on *C. albicans* involves its inhibitory activity on folic acid metabolism [148]. This phenomenon may elucidate the molecular mechanism behind the synergy observed between EGCG and azole antifungals. Evensen and Braun reported that the inhibition of biofilm formation and maintenance of *C. albicans* is expedited through the impairment of proteasomal activity caused by EGCG, leading to cellular metabolic and structural disruptions [151].

Tyrosol is a dietary phenolic compound present in virgin olive oil and wine and also a quorum-sensing molecule in *C. albicans* which stimulates germ-tube formation and yeast-to-hypha transition during biofilm formation [97]. This compound may have a significant impact on preventing diseases like cancer and metabolic, neurological, and cardiovascular disorders [152]. Its use against fungal infections has also been proposed. Cordeiro and colleagues stated that exogenous tyrosol significantly reduces the biofilm formation of clinical isolated *C. albicans* and *C. tropicalis* and showed that, when combined with amphotericin B (52.5 and 90 μg/mL, respectively), tyrosol at a concentration of 187.5 μM reduced *C. albicans* biofilm formation by 69% and *C. tropicalis* biofilm formation by 63%. The capacity to inhibit the mature biofilms of both *C. albicans* and *C. tropicalis* was found to be markedly lower (36.5% and 36.4%, respectively, at the same concentrations used to investigate antibiofilm formation properties) [153]. Fluconazole (3490 μg/mL) and itraconazole (1020 μg/mL) combined with 187.5 μM of tyrosol inhibited *C. albicans* biofilm formation by 55% and 70%, respectively. However, at the same concentration, these combinations had no activity against mature biofilm. Similarly, fluconazole (4720 μg/mL) and tyrosol (225 μM) were not able to inhibit *C. tropicalis* mature biofilm, but they have been shown to be active against the biofilm formation at the same concentrations, with inhibition rates of 58 and 61% [153]. Monteiro and colleagues reported that tyrosol significantly decreased the number of adhered cells to the acrylic surface in both single and mixed cultures of *C. albicans* and *C. glabrata* [154].

The presence of Pseudolaric Acid A (PAA), a diterpenoid found in the pine bark of *Pseudolarix kaempferi*, has been reported to exert inhibitory effects on *C. albicans.* The pine bark of *Pseudolarix kaempferi* is used in traditional Chinese medicine to treat dermatomycosis. Zhu and colleagues have studied the possible use of PAA in association with fluconazole against *C. albicans* ATCC 90028. They showed that both PAA and fluconazole lead to a dose-dependent decrease in *C. albicans* biofilm formation after 1.5 h of incubation. They found that the inhibition rate was about 20% using 8 μg/mL PAA and 23% using 0.25 μg/mL fluconazole. PAA (4 μg/mL) in association with fluconazole (0.5 μg/mL) exhibited a 42% inhibition of biofilm formation in the early stage (1.5 h). PAA (4 μg/mL) in combination with fluconazole (0.5 μg/mL) reduced biofilm formation by 49%, after 6 h of formation [155]. During the mature stage of biofilm formation (24 h), neither PAA nor fluconazole individually exhibited substantial antibiofilm activity, even at the highest drug concentration of 512 μg/mL. However, when PAA (4 μg/mL) and fluconazole (0.5 μg/mL) were combined, there was a 58% reduction in biofilm formation at 24 h. Also, cell adhesion was inhibited by 44% with 4 μg/mL of PAA and 0.5 μg/mL of fluconazole. Zhu and colleagues demonstrated that when fluconazole (0.5 μg/mL) was combined with PAA (4 μg/mL), the numbers of both planktonic cells and hyphae were lower than those in the drug-free control. This suggests that the combination of PAA with fluconazole inhibited the yeast-to-hypha transition and hypha formation, consequently hindering biofilm formation. The yeast-to-hypha transition is an important process in fungi like *Candida*, where yeast is the predominant growth form, but the transition to a filamentous form is crucial for biofilm formation [155].

The pine bark of *Pseudolarix kaempferi* also contains another diterpene, Pseudolaric Acid B (PAB), also known for its inhibitory activity against fungi. Li and colleagues showed that the combination of PAB and fluconazole displayed inhibitory effects on both early and mature biofilms. After 6 h of incubation, the combination of 16 µg/mL of fluconazole and 2 µg/mL of PAB on *C. tropicalis* inhibits 80.36% of biofilm formation. In addition, 64 µg/mL of PAB and 2 µg/mL of fluconazole inhibits 50% of mature biofilm. Among the mechanisms of action of PAB, the authors reported that PAB inhibited spore germination and mycelium formation and caused damage to cell integrity, resulting in cell deformation, swelling, collapse, and perforation of the outer membrane [156].

Coumarins are a group of natural chemical compounds that include a wide range of molecules with a similar basic structure. These compounds are widely distributed in nature and are found in various plants, such as coumarin, which is present in some herbs and spices, including cinnamon and vanilla. Some coumarins have pharmacological and biological properties, such as antioxidant, anti-inflammatory, and other biological properties.

The combination of fluconazole with osthole, a natural coumarin derived from the *Cnidium monnieri* plant, has been investigated by Li and colleagues against *C. albicans* SC5314 biofilm formation. The authors demonstrated the synergism of fluconazole and osthole against the planktonic growth and against *C. albicans* biofilm formation. Osthole or fluconazole alone did not exhibit an antibiofilm effect. However, the combination of fluconazole and osthole had a significant synergistic effect by reducing the biofilm formation by 90% at a concentration of 8 μg/mL for both. When it comes to the mechanism of action, the authors’ experiments, as indicated by the results of an expression profile microarray, revealed significant changes in the expression of genes associated with oxidation–reduction processes, energy metabolism, or transportation following the combined treatment with fluconazole and osthole. From the microarray data, the authors observed that the expression of multidrug transporter genes exhibited an opposite trend following the combined treatment with fluconazole and osthole [157,158].

Essential oils are concentrated blends of terpenes and other aromatic compounds found in some plants. Many essential oils, such as tea tree oil, oregano oil, and cinnamon oil, are known for their antimicrobial properties [159,160,161,162]. These essential oils can be extracted from various parts of the plants, such as the leaves, flowers, roots, or fruits. 

The genus *Oreganum* produces essential oils enriched in carvacrol, eugenol, and thymol. It has been shown that the addition of terpenoids in combination with fluconazole prevented *C. albicans* biofilm growth in the early phase of formation. Carvacrol at a concentration of 62 μg/mL did not have an inhibitory activity, but it allowed the concentration of fluconazole needed for biofilm formation inhibition to be decreased to 32 μg/mL. At these concentrations, the synergism between carvacrol and fluconazole was confirmed with a FICI value of 0.311. The combination of 125 μg/mL of eugenol with 2 μg/mL of fluconazole also had a synergistic effect, with a FICI of 0.25 against biofilm formation, whereas no interaction was detected against mature biofilm (FICI > 1). The combination of thymol and fluconazole resulted in no synergistic interaction against both biofilm formation and mature biofilm. Furthermore, 500 μg/mL of carvacrol decreases the fluconazole dosage needed to inhibit mature biofilms by 32-fold, although no synergistic effects have been registered (FICI > 0.5). In the manuscript, the authors report that a study on *Saccharomyces cerevisiae* revealed that eugenol and carvacrol induce membrane disintegration, loss of ions, and interference in the TOR signalling pathway, ultimately leading to a loss in viability. Additionally, terpenoid-induced alterations in permeability and membrane fluidity result in the degradation of the cell wall, which impacts the adherence of *C. albicans* to solid surfaces, and the increased influx of fluconazole results in the inhibition of biofilm formation [121].

Farnesol is a chemical compound belonging to the sesquiterpene class, which are terpenes composed of three isoprene units. Farnesol is found in various plants and is responsible for their characteristic aroma. It is also used in perfumery and the cosmetics industry for its pleasant scent. Additionally, it has been studied for its potential biological properties, including possible antibacterial and anti-inflammatory effects. Many plants, including citronella, lemon grass, tuberose, cyclamen, rose, neroli, balsam, and musk, contain farnesol in their essential oils, and it is also an autoregulatory molecule produced by *C. albicans*, which inhibits biofilm formation and the yeast-to-hyphae transition. Farnesol is used to treat hyperlipidemia, atherosclerosis, diabetes, allergic asthma, and obesity [163]. Synergy between farnesol and amphotericin B has been investigated by Katragkou and colleagues [164]. When 14 μM of farnesol was combined with 1 μg/mL of amphotericin B, *C. albicans* SC5314, a well-characterised strain that forms biofilms, reduced its biofilm formation. The MIC value of farnesol decreased from 600 μM when used alone to 14 μM when combined with amphotericin B, and for amphotericin B, the MIC values decreased from 1.5 μg/mL (alone) to 1 μg/mL (in combination) with a FICI of 0.79. Katrgkou and his group calculated the FIC index of the combination farnesol–fluconazole against *C. albicans* SC5314. They obtained a synergistic effect (FICI = 0.5) at a concentration of 150 μM of farnesol and 64 μg/mL of fluconazole. The authors observed, concerning the mechanism of action of farnesol, structural distortion, with the biofilm showing a more sparse appearance compared to untreated (control) biofilms. This includes a loss of elongated hyphal elements and a predominance of yeast-like forms. Farnesol has been tested in combination with fluconazole, voriconazole, itraconazole, posaconazole, and isoconazole against *C. albicans* biofilm formation [164]. The combination posaconazole–farnesol was the most active against *C. albicans* biofilm formation, with SMIC50 at a concentration of 0.25 μg/mL and 4.69 μg/mL, respectively. At the same concentration, farnesol inhibited 50% of biofilm formation in combination with 0.5 μg/mL of itraconazole or isoconazole. In addition, 1 μg/mL of voriconazole was needed to reduce the biofilm by 50%, together with 4.69 μg/mL of farnesol. The combination of farnesol and fluconazole was less active, and a higher concentration of these compounds was necessary to inhibit 50% of biofilm inhibition (75 μg/mL and 64 μg/mL, respectively). The combination of farnesol with posaconazole resulted in the most active combination, with an SMIC50 of 2.34 μg/mL of farnesol and 0.25 μg/mL of posaconazole against *C. auris* biofilm formation. The highest concentrations needed were 75 μg/mL and 64 μg/mL for the combination of farnesol/fluconazole. The authors showed that exposure to farnesol significantly increased phospholipase activity in *C. albicans*. Farnesol induced the production of reactive species in a dose-dependent manner and enhanced resistance to oxidative stress in *C. albicans*. Additionally, due to its amphiphilic properties, farnesol could be incorporated into cell membranes, affecting their fluidity and integrity. Farnesol was found to influence cellular polarisation and membrane permeability in both *C. parapsilosis* and *C. dubliniensis* [165].

Gypenosides (Gyp) are a group of triterpenoid saponins that were isolated from the perennial creeping plant *Gynostemma pentaphyllum Makino*, which is mostly located in southwest China [166] and is used in traditional Chinese medicine. *Gynostemma pentaphyllum* extracts possess several pharmacological properties, such as anti-inflammatory, antioxidative, and anticancer properties [167,168]. The combination of fluconazole and Gyp, tested against two fluconazole-resistant clinical *C. albicans* strains, showed synergistic activity against biofilm in the early stage of formation (4 h). At 8 h and 12 h of incubation, the FICIs were > 0.5, indicating no synergistic activity. The authors showed that the co-administration of fluconazole and Gyp inhibited yeast-to-hyphal conversion. When Gyp and fluconazole were used individually, they each had a slight effect on reducing the density and length of the hyphae in *C. albicans* cells. Furthermore, Gyp demonstrated antifungal activity against fluconazole-resistant *C. albicans* by inhibiting efflux pump activity [166].

Asiatic Acid (2α,3β,23-trihydroxyursan-12-en-28-oic acid, AA) is an aglycone of pentacyclic triterpenoids of the ursane class. Mainly *Centella asiatica* contains this phytocompound, but it is present in a wide variety of different species, including *Prunella vulgaris*, *Nepeta hindostana*, and *Combretum nelsonii*. The effect of AA alone and the interactions of fluconazole and AA against *C. albicans* biofilms were tested by Wang and colleagues [169]. They found no synergistic activity between AA and fluconazole against both 4 h and 8 h biofilm, with a FICI > 0.5. However, the concentration of AA used in combination was 2-fold decreased, whereas the fluconazole concentration was decreased from 64 μg/mL to 0.125 μg/mL and 0.25 μg/mL for 4 h and 8 h biofilm, respectively. The authors stated that the synergistic effect between AA and fluconazole could be linked to AA’s impact on the efflux pump in resistant *C. albicans.* Both AA alone and in combination with fluconazole exhibited antifungal activity, possibly through the induction of intracellular reactive oxygen species accumulation. Additionally, the combination of AA and fluconazole inhibited the hyphal growth of *C. albicans*, which could represent one of the mechanisms underlying the synergistic effect against *C. albicans* [169].

Cabbage, broccoli, horseradish, Brussels sprouts, and mustard belong to the family Cruciferae and contain isothiocyanates (ITCs). Their presence is considered responsible for excellent chemotherapeutic properties [170]. ITCs, including allyl isothiocyanate (AITC), have potential anticancer and antibacterial effects [171]. Several studies have shown the activities of AITC against foodborne bacterial pathogens and their biofilm and pathogenic fungi [172,173,174,175]. In a study conducted by Raut and colleagues, the anti-*Candida* biofilm activity of AITC was investigated, both alone and in combination with fluconazole. AITC alone showed a dose-dependent capacity to inhibit early biofilm development. AITC alone at 500 μg/mL inhibited more than 70% of biofilm formation of *C. albicans* ATCC 90028, and 512 μg/mL of fluconazole was needed to obtain *C. albicans* biofilm reduction. Due to the combination, the concentrations needed for both AITC and fluconazole decreased. The combination of 125 μg/mL of AITC and 4 μg/mL of fluconazole inhibited the biofilm formation by *C. albicans* [176]. The fluconazole concentration decreased by 128-fold when used in combination with AITC against biofilm formation with a FICI of 0.257. The AITC–fluconazole combination was synergistic also against the preformed biofilms, with an inhibition rate of 50% at the combination of 250 μg/mL of AITC and 32 μg/mL of fluconazole. The FICI values against the preformed biofilms of *C. albicans* ATCC 90028 and *C. albicans* GMC 03 were 0.265 and 0.312, respectively. In terms of the mechanism of action, the authors demonstrated that AITC interfered with serum-induced yeast-to-hypha morphogenesis in a concentration-dependent manner [176].

N-butylphthalide (NBP) is a natural compound derived from celery seeds (*Apium graveolens*) that has garnered significant interest in the field of medicine for its diverse range of potential therapeutic properties, including neuroprotection, cardiovascular benefits, antioxidant capabilities, and anti-inflammatory effects. In a study evaluating the use of N-butylphthalide and fluconazole in combination against clinical strains of *C. albicans*, the results showed that N-butylphthalide exhibited antibiofilm activity against biofilms preformed in less than 12 h. The combination of N-butylphthalide and fluconazole demonstrated synergistic interactions against some clinical isolates with preformed biofilms at various time points (4 h, 8 h, and 12 h). However, one clinical isolate displayed resistance, as its 24 h biofilm was not sensitive to the highest concentrations of both N-butylphthalide and fluconazole, whether used individually or in combination. Overall, the authors assert that N-butylphthalide exhibited potential in addressing biofilms; however, its efficacy fluctuated based on the isolate and biofilm maturity, with limited instances of synergistic effects being observed. In terms of mechanism of action, the authors observed that at a concentration of 64 μg/mL, N-butylphthalide induced the formation of loose and patchy hyphae. However, as the concentration increased to 128 μg/mL, the cells mainly remained in the yeast form, with only a few filaments visible in the field of vision. The results also showed that N-butylphthalide caused a significant dose-dependent accumulation of intracellular ROS in *C. albicans*. Furthermore, N-butylphthalide significantly enhanced drug absorption after 20 min, with cells in the N-butylphthalide-treated group absorbing higher concentrations of Rh6G compared to the control group [177].

Palmatine is an isoquinoline alkaloid present in *Rhizoma Coptidis* and *Mahonia aquifolium*. Several studies have elucidated its potential medicinal applications attributable to its pharmacological and biochemical characteristics. Palmatine is effective against bacterial, viral, and fungal infections as well as jaundice, diarrhoea, hypertension, and inflammation [178,179,180]. Wang and colleagues investigated the antibiofilm activity of palmatine, alone and in combination with fluconazole or itraconazole, against *C. albicans*, *C. parapsilosis*, *C. tropicalis*, *C. glabrata*, *C. krusei*, and *C. guilliermondii* [181]. *C. parapsilosis* was the only sensitive strain to palmatine, fluconazole, and itraconazole used alone. The concentrations needed to obtain 80% biofilm inhibition dramatically decreased when used in combination. For the different strains, the SMIC80 of the combination palmatine–fluconazole ranged from 8 μg/mL to 256 μg/mL for palmatine and from 64 μg/mL to 256 μg/mL for fluconazole. A clear decrease in the concentrations was shown when the compounds were used in combination, and synergistic effects were observed against all the tested strains, with FICI values ranging from 0.125 to 0.375, with *C. guilliermondii* ATCC 6260 and *C. tropicalis* GDM2.147 being two of the most sensitive strains [169]. The palmatine–itraconazole combination resulted in an indifferent interaction only when tested against *C. glabrata* ATCC 2340. The authors demonstrated that, following treatment with palmatine and fluconazole/itraconazole, there was a significant reduction in hyphal and blast conidia cells. Additionally, the concurrent administration of palmatine and fluconazole altered the normal function of efflux pumps in the *Candida* species tested [181].

Harmine hydrochloride (HMH) is a compound derived from the Chinese traditional herb harmel (*Peganum harmala*). It is traditionally used in Chinese herbal medicine for its antitussive properties, primarily as a remedy for cough relief [182]. HMH is an alkaloid compound with potential pharmacological effects. While it is traditionally known for its cough-relief properties, it has also garnered interest for its neuropharmacological effects, potential antioxidant activity, and applications beyond respiratory issues. The antibiofilm synergism of HMH combined with three azoles on resistant *C. albicans* clinical strains has been evaluated by Li and colleagues [183]. When used alone, HMH, fluconazole, itraconazole, and voriconazole have been shown to be ineffective against *C. albicans* adhesion and biofilm formation. HMH in combination with fluconazole, itraconazole, and voriconazole inhibited *C. albicans* adhesion with FICI values < 0.5. Regarding the mechanism of action, the authors demonstrated that the drug combination significantly elevates the intracellular concentration of [Ca^2+^] and the accumulation of intracellular ROS. Furthermore, the activity of metacaspases was substantially enhanced by the drug combination, thereby triggering the apoptotic process [183].

Medicinal plants such as *Hydrastis canadensis, Coptis chinensis, Berberis aquifolium, Berberis vulgaris* (barberry), *and Berberis aristata* contain the alkaloid berberine [184]. Plants produce berberine as a defence molecule to protect them against microorganisms. Several studies have investigated its antimicrobial activity against bacteria, fungi, protozoans, viruses, and helminths [185]. Wei and his group investigated the combination of berberine and miconazole using a dual-flow cell model. In this model, *C. albicans* SC5314 cells were allowed to attach to glass disk surfaces, preconditioned with artificial saliva. Glass discs supplied the right background for dark-field microscopy to visualise the unstained fungal biofilms. When tested individually, miconazole at 16 μg/mL and berberine at 0.8 μg/mL did not inhibit the formation of *C. albicans* biofilm. However, a significant 91% inhibition of biofilm formation was observed when the combination of berberine and miconazole was used after 24 h [186].

**Figure 2:**
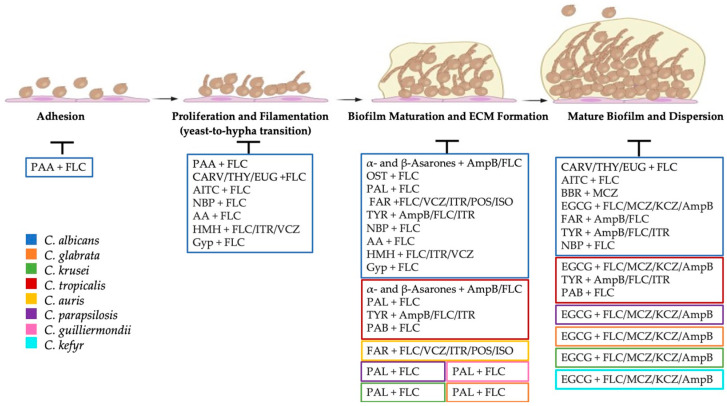
Activity of plant compounds in combination with antimicrobial drugs on different steps of *Candida* biofilm formation. Harmine hydrochloride (HMH), tyrosol (TYR), Pseudolaric Acid A (PAA), Pseudolaric Acid B (PAB), Asiatic Acid (AA), gypenosides (Gyp), epigallocatechin gallate (EGCG), palmatine (PAL), berberine (BBR), allyl isothiocyanate (AITC), osthole (OST), carvacrol (CARV), eugenol (EUG), thymol (THY), farnesol (FAR), N-butylphthalide (NBP), amphotericin B (AmpB), fluconazole (FLC), itraconazole (ITR), voriconazole (VCZ), posaconazole (POS), isoconazole (ISO), miconazole (MCZ), ketoconazole (KCZ).

Berberine was discovered to inhibit key enzymes (sterol 24-methyl transferase and chitin synthase) in the ergosterol and chitin biosynthesis pathways, resulting in the enhanced permeability of cell membranes and cell walls in *C. albicans* [186] (Table 2; Figure 2).

## 8. Synergy between Antimicrobial Chemotherapeutics and Natural Compounds against Polymicrobial Biofilms

Polymicrobial biofilms, composed of diverse microorganisms embedded within an extracellular polymeric substance matrix, present a significant challenge in various settings. These communities are characterised by complex interactions among microorganisms. Among polymicrobial biofilms, those involving *S.aureus*, *Candida* species, and *P. aeruginosa* have garnered attention due to their clinical relevance.

Polymicrobial biofilms formed by *S. aureus* and *Candida* spp. are frequently encountered in healthcare settings, often on medical devices. This interplay between bacteria and fungi can exacerbate infections and hinder treatments.

The combination of *S. aureus* and *P. aeruginosa* in biofilms is clinically significant, especially in chronic wounds, respiratory infections, and cystic fibrosis lung infections. These biofilms exhibit enhanced resistance to antibiotics and host immune responses.

Polymicrobial biofilms involving *P. aeruginosa* and *Candida* spp. have been observed in various contexts. They can lead to challenging clinical scenarios by combining *Pseudomonas*’ virulence factors with *Candida*’s adaptability.

The differences between monomicrobial and polymicrobial biofilms lie in their complexity and the interactions among multiple species. Understanding these intricate microbial communities is essential for improving clinical outcomes and addressing the challenges posed by biofilm-related infections.

Treating polymicrobial biofilms poses a formidable challenge due to the intricate interactions among multiple microbial species. These biofilms often exhibit heightened resistance to conventional antimicrobial agents and immune responses. To combat this issue, some researchers have explored the potential of combining antimicrobial drugs with plant-derived compounds. However, it is worth nothing that only a limited number of studies have delved into this area, investigating the activity of such combinations. 

Jafri and Ahmad documented the antibiofilm properties of thymol and eugenol from essential oils, both by themselves and in conjunction with vancomycin and fluconazole, against mixed biofilms of *C. albicans* and *S. aureus*. The highest synergy was found between thymol and vancomycin (FICI = 0.125), highlighting the synergy between phytocompounds and antimicrobial medications in varied combinations. Additional FICI values were also recorded for various combinations [187].

Gao et al. investigated the combination of amphotericin B and berberine for its activity against the formation of dual-species biofilms formed by *C. albicans* and *S. aureus*. In this study, *C. albicans* SC5314 and two different *S. aureus* strains (ATCC 25923 and the clinical *S. aureus* strain HNS0029) were utilised. The combination of amphotericin B and berberine resulted in a reduction of over 60% in the biomass of the dual-species biofilm formed by *C. albicans* and *S. aureus* HNS0029. Similarly, in dual-species biofilms formed by *C. albicans* and *S. aureus* ATCC 25923, the combination of the two agents at the same concentrations led to a reduction of 69% [188].

The study of Tan and colleagues assessed the combined effects of 2-aminobenzimidazole and curcumin on *C. albicans* and *S. aureus* mixed species biofilm formation and preformed biofilms. The combination exhibited enhanced efficacy against mixed biofilms compared to monotherapy [189] (Table 3).

## 9. Conclusions

Microbial pathogens living in biofilms are much more resistant to antibiotics and to elimination by the immune system, which has promoted the search for new strategies to control biofilm infections. Many researchers have combined plant compounds with antimicrobial activity with antibiotics or chemotherapeutics. Encouraging results were observed with EGCG in combination with different antimicrobials towards *P. aeruginosa*, *S. aureus*, and all species belonging to the genus *Candida*. These combinations are interesting and raise great expectations, but it must also be borne in mind that for many compounds the mechanism of action is still unknown and that these drugs influence each other and complicate pharmacokinetics. It is also necessary for activities to be standardised to make it possible to compare results, which is often difficult to do. It is certainly necessary for these studies to continue in order to ensure the correct use of combinations and to also make systemic administration possible. To date, one might suggest the topical use of the most active combinations to resolve muco-cutaneous infections, such as chronic wounds.

## Figures and Tables

**Table 1:** Synergism between plant compounds and antibacterial agents against *Staphylococcus aureus* and *Pseudomonas aeruginosa*.

Plant Compounds (1)	Drugs (2)	Strains	Preformed Biofilm				Biofilm Formation				Reference
		*S. aureus*	1 (µg/mL)	2 (µg/mL)	Inhibition (%)	FICI ^1^	1 (µg/mL)	2 (µg/mL)	Inhibition (%)	^1^ FICI	
Epigallocatechin-3- gallate-stearate	Tetracycline	ATCC 14990 CRM-6538					200	15	94		[125]
Baicalein	Vancomycin	17546 (t037)	32	4							[127]
Mallotojaponin B	Chloramphe- nicol	ATCC 33591	1.56	3.9	73.97		1.56	3.9	50	0.393	[128]
		*P. aeruginosa*	1 (µg/mL)	2 (µg/mL)	Inhibition (%)	FICI ^1^	1 (µg/mL)	2 (µg/mL)	Inhibition (%)	FICI	
Zingerone	Ciprofloxacin	PAO-1	10,000	0.06			10,000	0.06			[129]
14-alpha-lipoyl andrographolide	Azithromycin	PAO-1					269.4	8			[130]
14-alpha-lipoyl andrographolide	Gentamicin	PAO-1					269.4	1			[130]
14-alpha-lipoyl andrographolide	Ciprofloxacin	PAO-1					269.4	0.75			[130]
14-alpha-lipoyl andrographolide	Streptomycin	PAO-1					269.4	8			[130]
Rutin	Gentamicin	MTCC 2488					200	2.5	85	0.50	[131]
Quercetin	Amikacin	YU-V10, YU-V11, YU-V15, YU-V28, and PAO-1	125	0.5 to 128	>90		125	0.5 to 128	>90	0.25	[132]
Quercetin	Tobramycin	YU-V10, YU-V11, YU-V15, YU-V28 PAO-1	125	0.5 to 128	>90		125	0.5 to 128	>90	0.50	[132]
Epigallocatechin-3-gallate-stearate	Erythromycin	ATCC CRM-9027					100	15	95		[125]

^1^ FICI: Fractional Inhibitory Concentration Index.

**Table 2:** Synergism between plant compounds and antifungal agents against *Candida* spp.

Plant Compounds (1)	Drugs (2)	Strains	Preformed Biofilm				Biofilm Formation				Reference
		*C. albicans*	1 (µg/mL)	2 (µg/mL)	inhibition (%)	FICI ^2^	1 (µg/mL)	2 (µg/mL)	inhibition (%)	FICI ^2^	
α-Asarones from *Acorus calamusin* rhizomes	Amphotericin B	MTCC 277					8	0.03	36		[118]
α-Asarones from *Acorus calamusin* rhizomes	Fluconazole	MTCC 277					8	0.06	40		[118]
α-Asarones from *Acorus calamusin* rhizomes	Clotrimazole	MTCC 277					16	0.12	39		[118]
β-Asarones from *Acorus calamusin* rhizomes	Amphotericin B	MTCC 277					8	0.06	21		[118]
β-Asarones from *Acorus calamusin* rhizomes	Fluconazole	MTCC 277					2	0.03	16		[118]
β-Asarones from *Acorus calamusin* rhizomes	Clotrimazole	MTCC 277					4	0.06	23		[118]
Pseudolaric Acid A	Fluconazole	ATCC 90028	4	0.5	58		4	0.5	49		[155]
Allyl isothiocyanate	Fluconazole	ATCC 90028	250	16	50	0.265	125	4	50	0.257	[176]
Carvacrol	Fluconazole	ATCC 90028	500	32		0.516	62	32		0.311	[121]
Eugenol	Fluconazole	ATCC 90028	1000	2		1	125	2		0.25	[121]
Thymol	Fluconazole	ATCC 90028	2000	2		1.001	1000	2		1.003	[121]
osthole, coumarin derivative	Fluconazole	SC5314					8	8	90		[158]
Berberine	Miconazole	SC5314	250,000	7800	90	0.25	16,000	800	91		[186]
Palmatine	Fluconazole	SC5314					64	256	80	0.2813	[181]
Palmatine	Itraconazole	SC5314					64	128	80	0.127	[181]
Farnesol	Fluconazole	SC5314					75	64	50		[165]
Farnesol	Voriconazole	SC5314					4.69	1	50		[165]
Farnesol	Itraconazole	SC5314					4.69	0.5	50		[165]
Farnesol	Posaconazole	SC5314					4.69	0.25	50		[165]
Farnesol	Isoconazole	SC5314					4.69	0.5	50		[165]
Epigallocatechin gallate	Miconazole	SC5314					1500	400	90	0.31	[149]
Epigallocatechin gallate	Fluconazole	SC5314					3000	400	90	0.19	[149]
Epigallocatechin gallate	Amphotericin B	SC5314					3000	1.56	90	0.37	[149]
Farnesol	Fluconazole	SC5314	33.356	64		0.5					[164]
Farnesol	Amphotericin B	SC5314	3.313	1		0.79					[164]
Farnesol	Micafungin	SC5314	66.711	0.25		0.49					[164]
Tyrosol	Amphotericin B	Clinical isolate	25.9	52.5	36.5		25.9	52.5	69		[153]
Tyrosol	Fluconazole	Clinical isolate	25.9	3490	0		25.9	3490	55		[153]
Tyrosol	Itraconazole	Clinical isolate	25.9	1020	0		25.9	1020	70		[153]
Allyl isothiocyanate	Fluconazole	GMC 03	250	32	50	0.312	62	4	50	0.132	[176]
N-butylphthalide	Fluconazole	Clinical isolate 04	64	0.5	80	>0.5					[177]
N-butylphthalide	Fluconazole	Clinical isolate 04	64	0.5	80	>0.5					[177]
N-butylphthalide	Fluconazole	Clinical isolate 04	64	4	80	>0.5					[177]
Asiatic Acid	Fluconazole	Clinical isolate 04	32	0.25	80	0.504					[169]
Asiatic Acid	Fluconazole	Clinical isolate 08	32	0.25	80	0.504					[169]
N-butylphthalide	Fluconazole	Clinical isolate 10	32	0.5	80	0.25					[177]
N-butylphthalide	Fluconazole	Clinical isolate 10	32	4	80	0.26					[177]
N-butylphthalide	Fluconazole	Clinical isolate 10	64	8	80	0.27					[177]
Harmine hydrochloride	Fluconazole	Clinical isolate 10	>1024	>128	50	2	16	0.25	50	0.018	[183]
Harmine hydrochloride	Itraconazole	Clinical isolate 10	>1024	>64	50	2	64	0.125	50	0.064	[183]
Harmine hydrochloride	Voriconazole	Clinical isolate 10	>1024	>64	50	2	64	0.125	50	0.064	[183]
Gypenosides	Fluconazole	Clinical isolate 10	32	0.5	80	0.254					[166]
Asiatic Acid	Fluconazole	Clinical isolate 10	32	0.25	80	0.504					[169]
N-butylphthalide	Fluconazole	Clinical isolate 16	32	0.5	80	0.25					[177]
N-butylphthalide	Fluconazole	Clinical isolate 16	32	2	80	0.25					[177]
N-butylphthalide	Fluconazole	Clinical isolate 16	64	4	80	0.26					[177]
Harmine hydrochloride	Fluconazole	Clinical isolate 16	>1024	>128	50	2	16	0.25	50	0.018	[183]
Harmine hydrochloride	Itraconazole	Clinical isolate 16	>1024	>64	50	2	64	0.25	50	0.066	[183]
Harmine hydrochloride	Voriconazole	Clinical isolate 16	>1024	>64	50	2	64	0.125	50	0.064	[183]
Gypenosides	Fluconazole	Clinical isolate 16					32	1	80		[166]
Asiatic Acid	Fluconazole	Clinical isolate 16	32	0.25	80	0.504					[169]
Palmatine	Fluconazole	Clinical isolate 73044					64	256	80	0.375	[181]
Palmatine	Itraconazole	Clinical isolate 73044					16	256	80	1	[181]
Palmatine	Fluconazole	Clinical isolate Z2003					32	256	80	0.281	[181]
Palmatine	Itraconazole	Clinical isolate Z2003					32	128	80	0.094	[181]
Palmatine	Fluconazole	Clinical isolate Z1402					32	256	80	0.156	[181]
Palmatine	Itraconazole	Clinical isolate Z1402					64	256	80	0.129	[181]
Palmatine	Fluconazole	Clinical isolate Z1407					64	64	80	0.156	[181]
Palmatine	Itraconazole	Clinical isolate Z1407					32	32	80	0.281	[181]
Palmatine	Fluconazole	Clinical isolates Z826					64	256	80	0.312	[181]
Palmatine	Itraconazole	Clinical isolates Z826					64	128	80	0.187	[181]
Palmatine	Fluconazole	Clinical isolate Z1309					32	128	80	0.312	[181]
Palmatine	Itraconazole	Clinical isolate Z1309					32	64	80	0.266	[181]
Epigallocatechin 3-O-gallate	Fluconazole	CDC 27974					62.5	32	80	0.563	[150]
Epigallocatechin 3-O-gallate	Ketoconazole	CDC 27974					31.25	32	80	0.313	[150]
Epigallocatechin 3-O-gallate	Fluconazole	CDC 28304					31.25	32	80	0.375	[150]
Epigallocatechin 3-O-gallate	Ketoconazole	CDC 28304					31.25	16	80	0.313	[150]
Epigallocatechin 3-O-gallate	Fluconazole	ATCC 24433					31.25	16	80	0.531	[150]
Epigallocatechin 3-O-gallate	Ketoconazole	ATCC 24433					31.25	8	80	0.281	[150]
Epigallocatechin gallate	Miconazole	ATCC 10231					375	50	90	0.16	[149]
Epigallocatechin gallate	Fluconazole	ATCC 10231					375	800	90	0.28	[149]
Epigallocatechin gallate	Amphotericin B	ATCC 10231					750	0.13	90	0.19	[149]
Plant compounds	Drugs	*C. parapsilosis*	1 (µg/mL)	2 (µg/mL)	inhibition (%)	FICI ^2^	1 (µg/mL)	2 (µg/mL)	inhibition (%)	FICI ^2^	
Palmatine	Fluconazole	ATCC 22019					32	0.0625	80	0.281	[181]
Palmatine	Itraconazole	ATCC 22019					8	128	80	0.156	[181]
Epigallocatechin gallate	Miconazole	ATCC 22019					6000	800	90	0.5	[149]
Epigallocatechin gallate	Fluconazole	ATCC 22019					1500	1600	90	0.56	[149]
Epigallocatechin gallate	Amphotericin B	ATCC 22019					375	1.56	90	0.27	[149]
		*C. tropicalis*	1 (µg/mL)	2 (µg/mL)	inhibition (%)	FICI ^2^	1 (µg/mL)	2 (µg/mL)	inhibition (%)	FICI ^2^	
α-Asarones from *Acorus calamusin* rhizomes	Amphotericin B	MTCC 184					4	0.25	39		[118]
α-Asarones from *Acorus calamusin* rhizomes	Fluconazole	MTCC 184					16	0.12	50		[118]
α-Asarones from *Acorus calamusin* rhizomes	Clotrimazole	MTCC 184					8	0.25	41		[118]
β-Asarones from *Acorus calamusin* rhizomes	Amphotericin B	MTCC 184					1	0.03	21		[118]
β-Asarones from *Acorus calamusin* rhizomes	Fluconazole	MTCC 184					2	0.03	16		[118]
β-Asarones from *Acorus calamusin* rhizomes	Clotrimazole	MTCC 184					2	0.06	23		[118]
Tyrosol	Amphotericin B	Clinical isolate	311	90	36,4		311	90	63		[153]
Tyrosol	Fluconazole	Clinical isolate	311	4720	0		311	4720	58		[153]
Tyrosol	Itraconazole	Clinical isolate	311	1045	0		311	1045	61		[153]
Palmatine	Fluconazole	Clinical isolate GDM 2.147					8	256	80	0.125	[181]
Palmatine	Itraconazole	Clinical isolate GDM 2.147					16	32	80	0.062	[181]
Epigallocatechin gallate	Miconazole	ATCC 13803					1500	800	90	0.31	[149]
Epigallocatechin gallate	Fluconazole	ATCC 13803					1500	400	90	0.19	[149]
Epigallocatechin gallate	Amphotericin B	ATCC 13803					3000	0.19	90	0.19	[149]
Pseudolaric Acid B	Fluconazole	ATCC 750	64	2	50		2	16	80.36		[156]
		*C. krusei*	1 (µg/mL)	2 (µg/mL)	inhibition (%)	FICI ^2^	1 (µg/mL)	2 (µg/mL)	inhibition (%)	FICI ^2^	
Palmatine	Fluconazole	ATCC 1182					8	128	80	0.281	[181]
Palmatine	Itraconazole	ATCC 1182					128	4	80	0.312	[181]
Epigallocatechin gallate	Miconazole	ATCC 14243					375	0.20	90	0.14	[149]
Epigallocatechin gallate	Fluconazole	ATCC 14243					1500	39.06	90	0.75	[149]
Epigallocatechin gallate	Amphotericin B	ATCC 14243					187.5	0.16	90	0.31	[149]
		*C. glabrata*	1 (µg/mL)	2 (µg/mL)	inhibition (%)	FICI ^2^	1 (µg/mL)	2 (µg/mL)	inhibition (%)	FICI ^2^	
Palmatine	Fluconazole	ATCC 2340					256	128	80	0.312	[181]
Palmatine	Itraconazole	ATCC 2340					512	512	80	0.187	[181]
Epigallocatechin 3-O-gallate	Fluconazole	CDC 27845					15.63	16	80	0.375	[150]
Epigallocatechin 3-O-gallate	Ketoconazole	CDC 27845					15.63	8	80	0.375	[150]
Epigallocatechin 3-O-gallate	Fluconazole	CDC 28398					15.63	16	80	0.313	[150]
Epigallocatechin 3-O-gallate	Ketoconazole	CDC 28398					15.63	16	80	0.313	[150]
Epigallocatechin 3-O-gallate	Fluconazole	ATCC 15126					15.63	8	80	0.281	[150]
Epigallocatechin 3-O-gallate	Ketoconazole	ATCC 15126					15.63	8	80	0.281	[150]
Epigallocatechin gallate	Miconazole	ATCC 66032					1500	200	90	0.31	[149]
Epigallocatechin gallate	Fluconazole	ATCC 66032					6000	400	90	0.5	[149]
Epigallocatechin gallate	Amphotericin B	ATCC 66032					1500	0.63	90	0.31	[149]
		*C. auris*	1 (µg/mL)	2 (µg/mL)	inhibition (%)	FICI ^2^	1 (µg/mL)	2 (µg/mL)	inhibition (%)	FICI ^2^	
Farnesol	Fluconazole	Clinical isolate 10					75	64	50		[165]
Farnesol	Itraconazole	Clinical isolate 10					4.69	0.5	50		[165]
Farnesol	Posaconazole	Clinical isolate 10					2.34	0.25	50		[165]
Farnesol	Isoconazole	Clinical isolate 10					9.375	0.125	50		[165]
Farnesol	Fluconazole	Clinical isolate 12					75	64	50		[165]
Farnesol	Voriconazole	Clinical isolate 12					4.69	0.5	50		[165]
Farnesol	Itraconazole	Clinical isolate 12					9.375	0.5	50		[165]
Farnesol	Posaconazole	Clinical isolate 12					2.34	0.25	50		[165]
Farnesol	Isoconazole	Clinical isolate 12					18.75	0.125	50		[165]
Farnesol	Fluconazole	Clinical isolate 27					75	64	50		[165]
Farnesol	Voriconazole	Clinical isolate 27					9.375	0.5	50		[165]
Farnesol	Itraconazole	Clinical isolate 27					9.375	0.5	50		[165]
Farnesol	Posaconazole	Clinical isolate 27					2.34	0.25	50		[165]
Farnesol	Isoconazole	Clinical isolate 27					9.38	0.125	50		[165]
		*C. guilliermondi*	1 (µg/mL)	2 (µg/mL)	inhibition (%)	FICI ^2^	1 (µg/mL)	2 (µg/mL)	inhibition (%)	FICI ^2^	
Palmatine	Fluconazole	ATCC 6260					32	256	80	0.156	[181]
Palmatine	Itraconazole	ATCC 6260					2	64	80	0.094	[181]
		*C. dubliniensis*	1 (µg/mL)	2 (µg/mL)	inhibition (%)	FICI ^2^	1 (µg/mL)	2 (µg/mL)	inhibition (%)	FICI ^2^	
Epigallocatechin 3-O-gallate	Fluconazole	CDC 27963					15.63	16	80	0.313	[150]
Epigallocatechin 3-O-gallate	Ketoconazole	CDC 27963					15.63	16	80	0.313	[150]
Epigallocatechin 3-O-gallate	Fluconazole	CDC 28551					15.63	16	80	0.375	[150]
Epigallocatechin 3-O-gallate	Ketoconazole	CDC 28551					15.63	8	80	0.375	[150]
		*C. kefir*	1 (µg/mL)	2 (µg/mL)	inhibition (%)	FICI ^2^	1 (µg/mL)	2 (µg/mL)	inhibition (%)	FICI ^2^	
Epigallocatechin gallate	Miconazole	ATCC 46764					750	100	90	0.63	[149]
Epigallocatechin gallate	Fluconazole	ATCC 46764					750	200	90	0.63	[149]

^2^ FICI: Fractional Inhibitory Concentration Index.

**Table 3:** Synergism between plant compounds and antimicrobial agents against polymicrobial biofilm.

Plant Compounds (1)	Drugs (2)	Strains	Preformed Biofilm				Biofilm Formation				Reference
			1 (µg/mL)	2 (µg/mL)	Inhibition (%)	FICI	1 (µg/mL)	2 (µg/mL)	Inhibition (%)	FICI ^3^	
Thymol	Fluconazole	*C. albicans* J-01					3.25	32	80	0.250	[187]
		*S. aureus* MTCC3160									
Eugenol	Fluconazole	*C. albicans* J-01					12.5	512	80	0.531	[187]
		*S. aureus* MTCC3160									
Thymol	Vancomycin	*C. albicans* J-01					3.25	128	80	0.125	[187]
		*S. aureus* MTCC3160									
Eugenol	Vancomycin	*C. albicans* J-01					12.5	512	80	0.281	[187]
		*S. aureus* MTCC3160									
Thymol	Fluconazole	*C. albicans* J-12					3.12	256	80	0.187	[187]
		*S. aureus* MTCC3160									
Eugenol	Fluconazole	*C. albicans* J-12					12.5	1024	80	0.562	[187]
		*S. aureus* MTCC3160									
Thymol	Vancomycin	*C. albicans* J-12					3.12	128	80	0.125	[187]
		*S. aureus* MTCC3160									
Eugenol	Vancomycin	*C. albicans* J-12					50	256	80	0.375	[187]
		*S. aureus* MTCC3160									
Berberine	Amphotericin B	*C. albicans* SC5314					128	4	60		[188]
		*S. aureus* HNS0029									
Berberine	Amphotericin B	*C. albicans* SC5314					128	4	69		[188]
		*S. aureus* ATCC 25923									
Curcumin	2-aminobenzimidazole	*C. albicans* DAY185	200	200	73.3		100	100	97.6		[189]
		*S. aureus * ATCC 6538									

^3^ FICI: Fractional Inhibitory Concentration Index.

## Data Availability

Data sharing is not applicable.
